# p53-induced RNA-binding protein ZMAT3 inhibits transcription of a hexokinase to suppress mitochondrial respiration

**DOI:** 10.1101/2025.05.12.653341

**Published:** 2025-05-13

**Authors:** Ravi Kumar, Simon Couly, Bruna R. Muys, Xiao Ling Li, Ioannis Grammatikakis, Ragini Singh, Mary Guest, Xinyu Wen, Wei Tang, Stefan Ambs, Lisa M. Jenkins, Erica C. Pehrsson, Raj Chari, Tsung-Ping Su, Ashish Lal

**Affiliations:** 1 Regulatory RNAs and Cancer Section, Genetics Branch, Center for Cancer Research (CCR), National Cancer Institute (NCI), National Institutes of Health (NIH), Bethesda, MD, USA; 2 Cellular Pathobiology Section, Integrative Neuroscience Branch, National Institute on Drug Abuse (NIDA), NIH, Baltimore, MD, USA; 3 Genome Modification Core, Frederick National Lab for Cancer Research, NCI, NIH, Frederick, MD, USA.; 4 Oncogenomics Section, Genetics Branch, CCR, NCI, NIH, Bethesda, MD, USA; 5 Laboratory of Human Carcinogenesis, CCR, NCI, NIH, Bethesda, MD, USA; 6 Mass Spectrometry Section, Laboratory of Cell Biology, CCR, NCI, NIH, Bethesda, MD, USA; 7 Advanced Biomedical Computational Science, Frederick National Laboratory for Cancer Research, Frederick, MD, USA

**Keywords:** p53, ZMAT3, HKDC1, JUN, RNA-binding protein, hexokinase, mitochondrial respiration, proliferation, proteomics, transcription, tumor suppressor, cancer

## Abstract

The tumor suppressor p53 is a transcription factor that controls the expression of hundreds of genes. Emerging evidence suggests that the p53-induced RNA-binding protein ZMAT3 is a key splicing regulator that functions in p53-dependent tumor suppression *in vitro* and *in vivo*. However, the mechanism by which ZMAT3 functions in the p53 pathway is largely unclear. Here, we discovered a function of ZMAT3 in inhibiting transcription of *HKDC1*, a hexokinase that regulates glucose metabolism and mitochondrial respiration. Using quantitative proteomics, we identified HKDC1 as the most significantly upregulated protein in *ZMAT3*-depleted colorectal cancer cells. *ZMAT3* depletion results in increased mitochondrial respiration that was rescued upon depletion of *HKDC1*, suggesting that HKDC1 is a critical downstream effector of *ZMAT3*. Unexpectedly, ZMAT3 did not bind to the *HKDC1* RNA or DNA but the identification of the ZMAT3-interactome uncovered its interaction with the oncogenic transcription factor JUN. ZMAT3 depletion resulted in increased JUN binding at the *HKDC1* promoter and increased *HKDC1* transcription that was rescued upon JUN depletion, suggesting that JUN activates *HKDC1* transcription in ZMAT3-depleted cells. Collectively, these data reveal a mechanism by which ZMAT3 regulates transcription and demonstrates that *HKDC1* is a key component of the ZMAT3-regulated transcriptome in the context of mitochondrial respiration regulation.

## Introduction

*TP53* is the most frequently mutated gene in human cancer and functions as a major tumor suppressor^1^. *TP53* mutations in the germline of Li Fraumeni patients and in sporadic cancer, are mostly missense mutations that occur in the DNA-binding domain resulting in loss of tumor suppressor function and in some cases, gain of oncogenic functions^2–4^. Deletion of the *Trp53* gene in mice results in spontaneous tumor development within 6 months of age at 100% penetrance, underscoring the importance of p53 as a tumor suppressor^5,6^. Mechanistically, p53 functions as a sequence-specific transcription factor activating the expression of hundreds of genes that control diverse cellular processes including but not limited to, cell cycle arrest, apoptosis, senescence and DNA repair^7,8^. Despite the undisputed role of p53 in tumor suppression, our understanding of how p53 target genes mediate the effects of p53 is not fully understood.

Among the p53 target genes, some such as *p21* (*CDKN1A*) control p53-dependent cell cycle arrest, whereas *PUMA* and *NOXA* are critical in inducing apoptosis downstream of p53^9–11^. *In vivo* studies in mice have demonstrated that *Abca1*, *Gls2*, *Mlh1*, *Padi4* and *Zmat3* play key roles in mediating the tumor suppressor effects of p53^12–15^. However, triple knockout mice lacking *p21*, *Puma* and *Noxa*, which are regulators of cell cycle arrest and apoptosis in the p53 pathway, do not develop tumors spontaneously, unlike p53 null mice^16^. This has led to search for new mechanisms and effectors of p53-mediated tumor suppression.

An emerging potent mediator of p53 is the p53 target gene *ZMAT3*, which functions as an RNA-binding protein and studies in mice strongly implicate *Zmat3* as a tumor suppressor^17–19^. Mechanistically, using transcriptome-wide approaches from crosslinked cells, we and others recently reported that ZMAT3 directly binds to intronic sequences in thousands of pre-mRNAs and regulates alternative splicing^17,20^. ZMAT3 has also been shown to interact with AU-rich elements in the 3′ untranslated regions (UTR) of target mRNAs either stabilizing its targets or promoting their decay^21–24^. A deeper understanding of the molecular mechanisms by which ZMAT3 functions is necessary to better understand how ZMAT3 functions in p53-mediated tumor suppression.

Here, to better understand the function of ZMAT3, we identified the ZMAT3-interactome and the proteins regulated by ZMAT3. Unexpectedly, this approach revealed that ZMAT3 inhibits mitochondrial respiration by interacting with the transcription factor JUN (c-Jun) to inhibit transcription of the hexokinase *HKDC1* (hexokinase domain containing 1). We chose to focus on *HKDC1* because by quantitative proteomics, HKDC1 was the most strongly up-regulated protein in ZMAT3-depleted colorectal cancer (CRC) cells. Hexokinases are the first rate-limiting enzymes in the glucose metabolic pathway that phosphorylate glucose to glucose-6-phosphate and thereby modulate glycolysis, oxidative phosphorylation, and the pentose phosphate pathway. There are four classic hexokinases (HK1–4), and recently HKDC1 was identified as the fifth hexokinase^25^. Besides its hexokinase function, HKDC1 also interacts with the mitochondrial membrane to maintain mitochondrial homeostasis and plays an important role in preventing cellular senescence^26–28^. Although, HKDC1 is reported to be overexpressed in many cancers and high HKDC1 expression is associated with poor clinical outcome^27–30^, how HKDC1 expression is regulated remains largely unclear. Our findings provide novel insights on the regulation and function of HKDC1 in the p53 pathway via transcriptional inhibition by a ZMAT3/JUN axis.

## Results

### The hexokinase HKDC1 is the most strongly upregulated protein in ZMAT3-knockout cells

**Figure 1.**
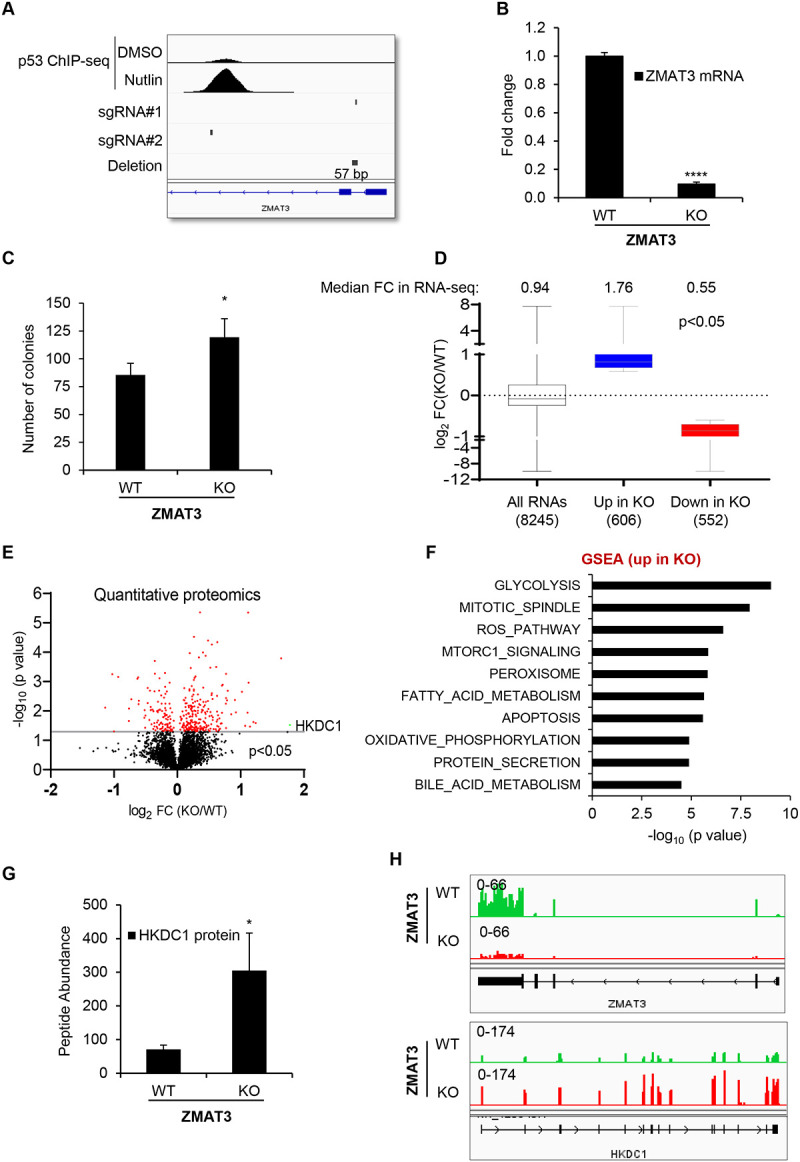
ZMAT3 depletion results in increased expression of genes related to glucose metabolism in colorectal cancer cells. **(A)** IGV snapshot shows location of the two sgRNAs used to generate *ZMAT3*-KO HCT116 cells, the observed 57 bp deletion near sgRNA#2 and the p53 ChIP-seq peak in the *ZMAT3* locus in response to p53 activation upon Nutlin treatment. The p53 ChIP-seq data was previously published^66^. **(B)** RT-qPCR was performed from 3 biological replicates of *ZMAT3*-WT and *ZMAT3*-KO HCT116 cells. *GAPDH* served as a housekeeping gene control. **(C)** Colony formation assays were performed from 3 biological replicates of *ZMAT3*-WT and *ZMAT3*-KO HCT116 cells. **(D)** Notched box plot of the log_2_FC in RNA abundance of differentially expressed genes after RNA-Seq analysis of *ZMAT3*-KO versus *ZMAT3*-WT HCT116 cells. Median values for each group are indicated at the top, and the number of RNAs for which data were obtained for each group is indicated at the bottom. **(E)** Volcano plots showing differentially expressed proteins (shown in red) identified by performing quantitative proteomics from *ZMAT3*-WT and *ZMAT3*-KO HCT116 cells. **(F)** Most significantly enriched pathways in the gene set enrichment analysis of significantly upregulated genes (p<0.05) from the *ZMAT3*-KO/*ZMAT3*-WT quantitative proteomics analysis. **(G)** TMT mass spectrometry peptide abundance of HKDC1 protein in *ZMAT3*-WT and *ZMAT3*-KO HCT116 cells. The values are the average of five biological replicates for ZMAT3-WT and four biological replicates for *ZMAT3*-KO cells. **(H)** IGV snapshot for ZMAT3 and HKDC1 from RNA-seq from *ZMAT3*-WT and *ZMAT3*-KO HCT116 cells. *p < 0.05, ****p < 0.0001.

To investigate the mechanism by which ZMAT3 promotes growth suppression, we utilized CRISPR/Cas9 to knockout *ZMAT3* in HCT116 cells (CRC) using two sgRNAs flanking the p53 response element in the second intron of the *ZMAT3*. Although this approach did not completely delete the region spanning the two sgRNAs, a ~57 bp region near sgRNA#1 was deleted resulting in >75% decrease in *ZMAT3* mRNA levels (Figure 1A and B). At the protein level, ZMAT3 was strongly induced only in *ZMAT3*-WT (wild-type) cells upon treatment with Nutlin, a small molecule that upregulates p53 and its target genes (Figure S1A). As expected, upon Nutlin treatment, p53 and its known target p21, were induced in both *ZMAT3*-WT and isogenic *ZMAT3*-KO (knockout) cells (Figure S1A). Depletion of ZMAT3 resulted in increased proliferation and clonogenicity, consistent with previous reports (Figure 1C and S1B)^18–20^.

To identify the genes regulated by ZMAT3, we next performed RNA-seq from biological triplicates of *ZMAT3*-WT and *ZMAT3*-KO HCT116 cells. As expected, *ZMAT3* mRNA levels markedly decreased (~7.5-fold) in *ZMAT3*-KO cells (Table S1); the recently identified direct ZMAT3 target gene *MDM4*^17^ was modestly but significantly upregulated in *ZMAT3*-KO cells (Table S1). Transcriptome-wide, upon loss of ZMAT3, 606 genes were significantly up-regulated (adj. p<0.05 and 1.5-fold change) and 552 were down-regulated with a median fold change of 1.76 and 0.55 for the up- and downregulated genes, respectively (Figure 1D and Table S1). Because we and others recently reported that ZMAT3 directly regulates alternative splicing^17,20^, we reasoned that ZMAT3-dependent changes in splicing could lead to altered protein levels without altering mRNA levels. We therefore performed quantitative proteomics from *ZMAT3*-WT and *ZMAT3*-KO HCT116 cells. At the protein level 228 proteins were significantly (p<0.05) up-regulated and 108 were down-regulated upon loss of ZMAT3 (Figure 1E) (Table S2).

ZMAT3 directly binds to intronic sequences in pre-mRNAs and can inhibit inclusion of the neighboring exon^17,20^. Depletion of ZMAT3 can therefore result in increased target gene expression. Gene set enrichment analysis (GSEA) for the proteins upregulated in *ZMAT3*-KO cells revealed glycolysis as the most significantly overrepresented biological process (Figure 1F). GSEA analysis from our RNA-seq data showed that glycolysis was among the top 10 overrepresented biological processes for the mRNAs upregulated in *ZMAT3*-KO cells (Figure S1D). Interestingly, the protein that was most strongly upregulated (~3.4-fold, p<0.05) in *ZMAT3*-KO cells was the hexokinase HKDC1 (Figure 1E, G and Table S2). The increase in HKDC1 expression was also observed at the mRNA level (Figure 1H, S1C and Table S1); as expected, *ZMAT3* mRNA levels were decreased in the *ZMAT3*-KO cells. We therefore chose to focus on *HKDC1* because it was the most strongly upregulated protein upon loss of ZMAT3 and is directly involved in regulating glucose metabolism and mitochondrial respiration that are cellular processes not been previously associated with ZMAT3.

### ZMAT3 inhibition of *HKDC1* expression is conserved and observed in diverse cell types

**Figure 2.**
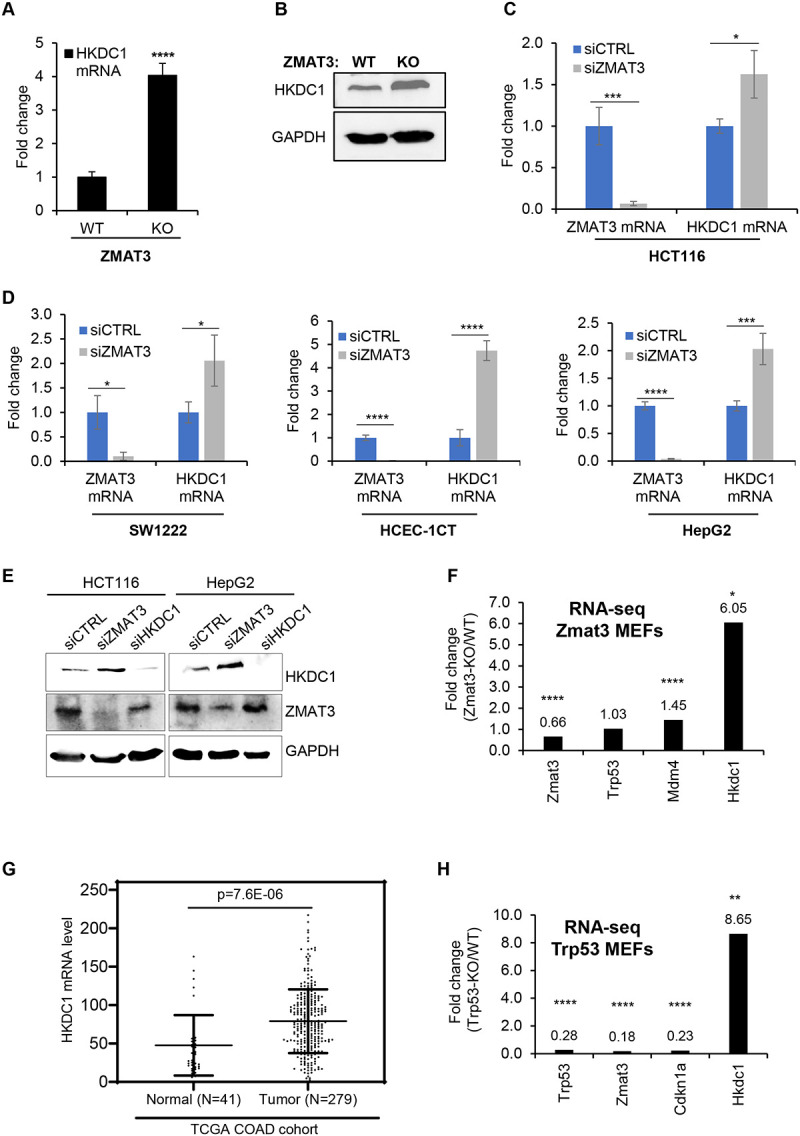
ZMAT3 negatively regulates HKDC1 expression in diverse cell types. **(A, B)** RT-qPCR and immunoblotting for HKDC1 from *ZMAT3*-WT and *ZMAT3*-KO HCT116 cells. GAPDH served as a housekeeping gene control. RT-qPCR are values are the average of three biological replicates. **(C, D)** RT-qPCR was performed in biological triplicates for *ZMAT3* and *HKDC1* mRNAs from HCT116, SW1222, HCEC-1CT and HEPG2 cells after transfection with a control (CTRL) siRNA or ZMAT3 siRNAs for 72 hr. GAPDH served as a housekeeping gene control. **(E)** Immunoblotting was performed for endogenous ZMAT3 and HKDC1 from HCT116 and HepG2 whole cell lysates after siRNA-mediated knockdown of ZMAT3 or HKDC1 for 72 hr. *GAPDH* served as housekeeping gene control. **(F)** Fold change for *Zmat3, Trp53, Mdm4* and *Hkdc1* mRNAs is shown from the RNA-seq from *Zmat3* knock-out and wild-type MEFs. (**G)** Analysis of *HKDC1* mRNA levels from normal colon tissues or CRC patient samples from the TCGA COAD cohort. N refers to the number of samples in each group. **(H)** Fold change *for Trp53, Zmat3, Cdkn1a* and *Hkdc1* mRNAs is shown from the RNA-seq from *Trp53* knock-out and wild-type MEFs. N refers to the number of samples in each group. *p < 0.05, ***p < 0.001, ****p < 0.0001

To validate inhibition of *HKDC1* expression by ZMAT3, we next performed RT-qPCR from *ZMAT3*-WT and *ZMAT3*-KO HCT116 cells. We observed ~4-fold upregulation of *HKDC1* mRNA upon ZMAT3 depletion (Figure 2A). The increase in HKDC1 expression was further confirmed at the protein level by immunoblotting (Figure 2B). Because these experiments were conducted from a single *ZMAT3*-KO clone, we next analyzed our recently published RNA-seq data^20^ conducted in biological triplicates from HCT116 cells transfected with a control siRNA (siCTRL) or ZMAT3 siRNAs (SMARTpool of 4 siRNAs). *ZMAT3* mRNA was strongly downregulated, and *HKDC1* was modestly but significantly up-regulated (Figure S2A and Table S3), a result that was validated by RT-qPCR (Figure 2C). GSEA analysis for the mRNAs up-regulated upon ZMAT3 knockdown showed that glycolysis was among the top 10 overrepresented biological processes (Figure S2B). Additionally, comparison of the RNA-seq data from the *ZMAT3*-WT vs *ZMAT3*-KO and CTRL siRNA vs ZMAT3 siRNA transfected HCT116 cells, indicated that 1023 genes were commonly up-regulated, and 1042 genes were commonly downregulated upon loss of ZMAT3 (Figure S2C and D), suggesting that ZMAT3 depletion results in altered expression of thousands of genes. GSEA analysis for the top 500 mRNAs up-regulated upon ZMAT3 knockdown showed that glycolysis was among the top 10 overrepresented biological processes (Figure S2E). Collectively, these data indicate that ZMAT3 inhibits the expression of genes that play important roles in glycolysis.

To determine if ZMAT3 inhibits *HKDC1* expression in multiple cell lines, we performed RT-qPCR after ZMAT3 knockdown in SW1222 (CRC cells), HCEC-1CT (immortalized human colonic epithelial cells), and HepG2 (liver cancer cells). We observed significant increase in *HKDC1* mRNA levels upon ZMAT3 knockdown in these cell lines (Figure 2D). At the protein level, we observed modest increase in HKDC1 levels upon knockdown of ZMAT3 in HCT116 and HepG2 cells (Figure 2E). Moreover, we explored recently published RNA-seq data from *Zmat3* knockout mouse embryonic fibroblasts (MEFs)^31^. *Hkdc1* mRNA was significantly upregulated (~6-fold) in *Zmat3*-KO MEFs (Figure 2F). And we also observed the expected upregulation of its target gene *Mdm4* while no changes in *Trp53* mRNA expression were observed.

In the context of human CRC, in the TCGA colorectal adenocarcinoma (COAD) cohort, *HKDC1* mRNA levels were significantly higher in tumors as compared to normal tissues (Figure 2G). Further analysis of the TCGA COAD data showed that as compared to p53 wild-type tumors, mutant p53 tumors exhibited significantly higher *HKDC1* mRNA levels (Figure 2G). As expected for the p53 target ZMAT3 and as shown previously in other cancer types^17^, ZMAT3 mRNA levels were significantly lower in the mutant p53 tumors as compared to p53 wild-type tumors (Figure S2F and G). Furthermore, RNA-seq from *Trp53* knockout MEFs from the same study mentioned above^31^ showed significant upregulation (~8.6-fold) of *Hkdc1* mRNA, and down-regulation of its target genes *Zmat3* and *Cdkn1a* (Figure 2H). These data suggest that *ZMAT3* and *HKDC1* mRNA expressions are negatively correlated in the p53 pathway and the inhibition of HKDC1 expression by ZMAT3 and p53 is conserved between human and mouse.

### ZMAT3 inhibits mitochondrial respiration by downregulating HKDC1

**Figure 3.**
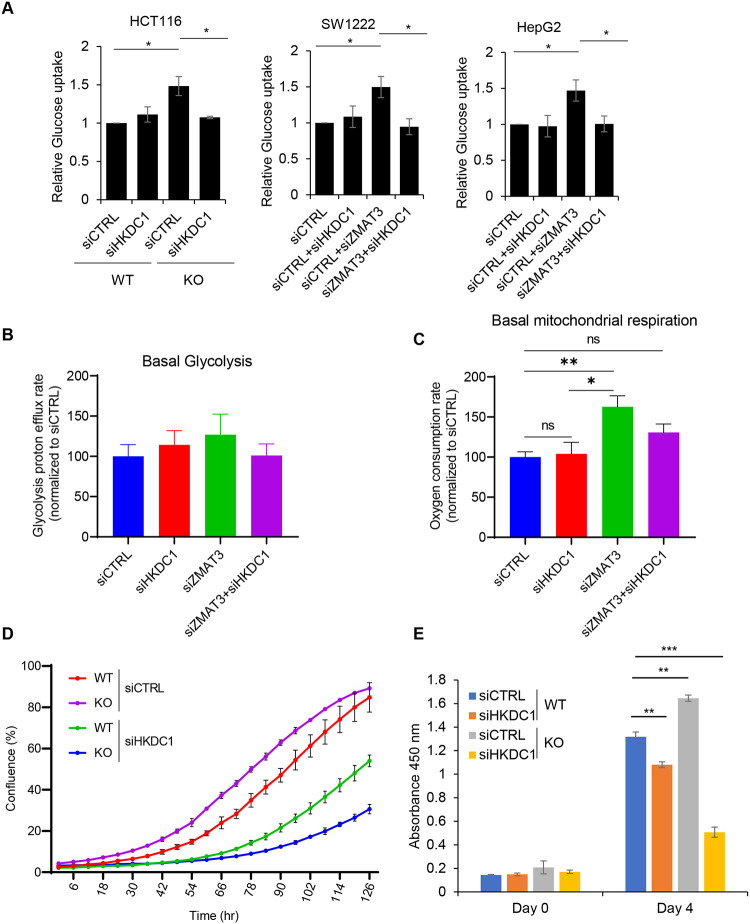
ZMAT3 inhibits mitochondrial respiration and proliferation via HKDC1. **(A)** 2-Deoxyglucose analog of glucose together with luminescence-based enzymatic assay was used to assess relative glucose uptake in *ZMAT3*-WT and *ZMAT3*-KO HCT116 cells in presence and absence of HKDC1. For SW122 and HEPG2 cells relative glucose was measured in the presence and absence of siRNA-mediated knockdown of HKDC1 and ZMAT3 alone or in combination. **(B, C)** Metabolic flux assays were performed for basal glycolysis rate and basal mitochondrial respiration rate in ZMAT3 and/or HKDC1 knockdown in HCT116 cells. (**D, E**) Incucyte live cell proliferation assays and CCK8-based cell proliferation in HCT116 *ZMAT3*-WT and KO cells in the presence and absence of siRNA-mediated knockdown HKDC1. *p < 0.05, (**) p < 0.01, (***) P<0.001. The results are the average of three independent experiments.

Previous studies suggest that HKDC1 plays a crucial role in regulating glucose metabolism and cell proliferation in various cell types^27,28^. To determine whether ZMAT3 regulates glucose metabolism and/or proliferation by regulating HKDC1 expression, we performed glucose metabolic assays. Since HKDC1 is a hexokinase and can regulate phosphorylation of glucose to glucose 6-phosphate when it enters the cells, we began with measuring changes in hexokinase activity upon ZMAT3 loss. We incubated the cells with the non-catabolic glucose analog 2-deoxy glucose (2-DG) for a short period of time and quantified the conversion of 2-DG to 2-DG6P using a luminescence-based assay. We observed a significant increase in relative 2-DG6P levels in *ZMAT3*-KO cells compared to WT cells. Interestingly, this increase was reversed when we knocked down HKDC1 (Figure 3A). Furthermore, siRNA-mediated knockdown of ZMAT3 in SW1222 and HEPG2 also showed a similar increase in hexokinase activity and the effect was reversed upon concurrent knockdown of ZMAT3 and HKDC1, suggesting that ZMAT3 inhibits glucose uptake, and this effect is HKDC1-dependent (Figure 3A). We then investigated whether increased hexokinase activity upon ZMAT3 knockdown leads to increased glycolysis in cells. To measure this, we performed Seahorse assays, measured extracellular acidification rate and calculated the glycolysis proton efflux. It should be noted that the proton efflux rate measured with Seahorse is reflecting the lactate production from glycolysis more than the pyruvate end point of glycolysis that fueled the mitochondria. Thus, we also measured mitochondria activity. Knockdown of ZMAT3 or HKDC1 by siRNAs resulted in modest increase in basal glycolysis but it was not significant (Figure 3B).

Recent studies suggest that HKDC1 plays a significant role in regulating mitochondrial respiration, and depletion of HKDC1 results in mitochondrial dysfunction and senescence^26,27^. We therefore determined a potential function of ZMAT3 in regulation of basal mitochondrial respiration by regulating HKDC1 expression. We conducted Seahorse assays, and to measure OCR (oxygen consumption rate) upon knockdown of HKDC1 or ZMAT3 or both. Interestingly, we found that ZMAT3 knockdown results in significantly increased basal mitochondrial respiration, and simultaneous ZMAT3 and HKDC1 knockdown rescues this effect (Figure 3C) and this, without affecting the oxygen consumption that could occur along cellular pathways that are not involving mitochondria. We did not observe any changes in non-mitochondrial respiration(Figure S3). Because these phenotypes can be associated with proliferation, we next asked whether HKDC1 regulates proliferation and whether it is an effector of ZMAT3. Live cell proliferation assays and CCK8 cell viability assays suggested that knocking down HKDC1 in both ZMAT3-WT and KO cells resulted in decreased cell proliferation. However, the effect of HKDC1 knockdown was more significant in *ZMAT3*-KO cells suggesting that HKDC1 plays a key role in promoting proliferation downstream of ZMAT3 (Figure 3D and E).

### The p53/ZMAT3 axis inhibits HKDC1 expression

**Figure 4.**
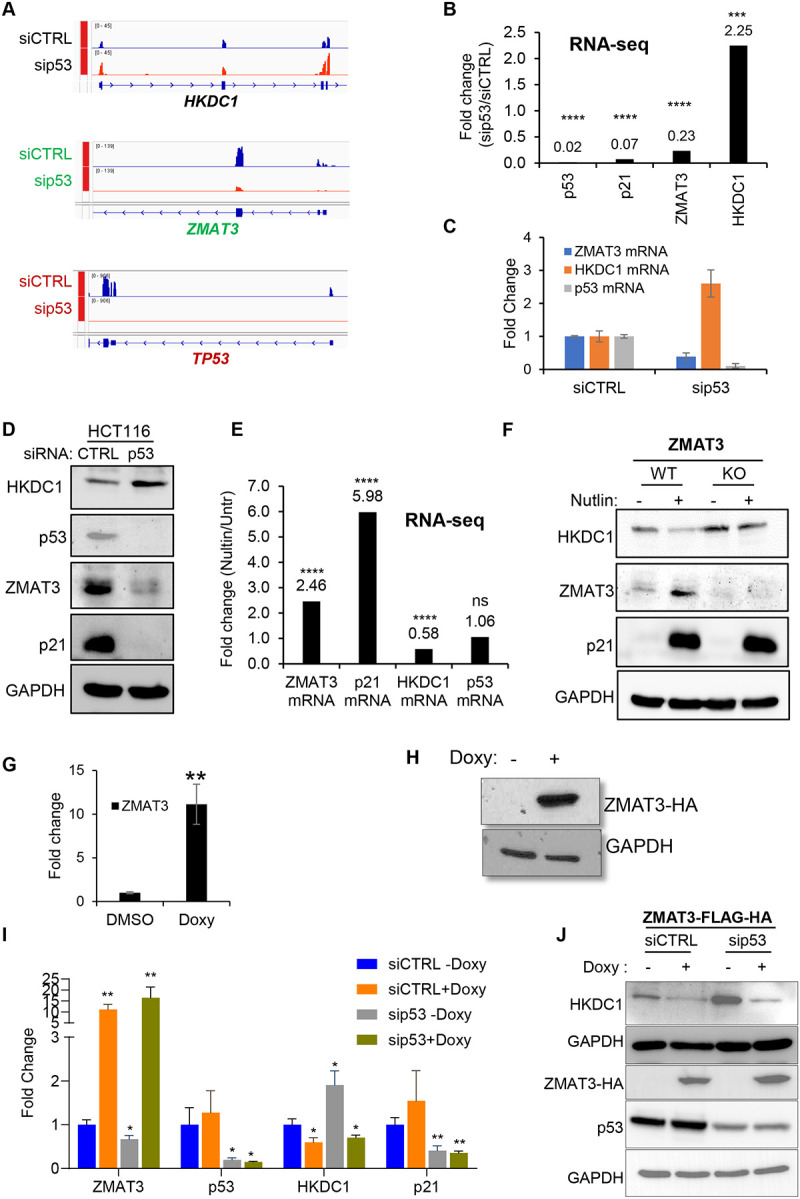
p53 negatively regulates HKDC1 expression in a ZMAT3-dependent manner. **(A)** IGV snapshots from the RNA-seq data following knockdown of p53 with p53 siRNAs. Data shows increased *HKDC1* mRNA levels and decreased *ZMAT3* mRNA levels upon p53 knockdown in HCT116 cells. **(B)** Fold change is shown for *p53, p21, ZMAT3*, and *HKDC1* mRNAs from the RNA-seq performed from siCTRL and sip53 transfected HCT116 cells. **(C, D)** HCT116 cells were transfected with CTRL siRNA or p53 siRNAs for 48 hr. The levels of *ZMAT3, p53*, and *HKDC1* mRNA or protein were measured by RT-qPCR (C) or immunoblotting from whole cell lysates (D). *GAPDH* was used as housekeeping gene control. **(E)** Fold change for ZMAT3, p21, HKDC1 and p53 mRNAs is shown from the RNA-seq from HCT116 cells treated with DMSO or Nutlin for 6 hr. **(F)** Immunoblotting was performed for HKDC1, ZMAT3 and p21 from *ZMAT3*-WT and *ZMAT3*-KO HCT116 cells with or without Nutlin treatment for 24 hr. GAPDH served as the loading control. ns refers to not significant. (**G,H**) Doxycycline inducible ZMAT3-FLAG-HA HCT116 cells treated with 2ug/ml doxycycline for 48 hr. The levels of ZMAT3 mRNA or protein induction were measured by RT-qPCR (G) or immunoblotting from whole cell lysates against HA antibody(H). *GAPDH* was used as housekeeping gene control. **(I, J)** Doxycycline inducible ZMAT3-FLAG-HA HCT116 cells were transfected with CTRL siRNA or p53 siRNAs for 48 hr followed by 48hr doxycycline treatment. The levels of *ZMAT3, p53*, and *HKDC1* mRNA or protein were measured by RT-qPCR (I) or immunoblotting from whole cell lysates (J). *GAPDH* was used as housekeeping gene control. *p < 0.05, **p < 0.01, ***p < 0.001, ****p < 0.0001

Because *ZMAT3* transcription is activated by p53, we next examined if the p53 pathway inhibits *HKDC1* expression. We therefore performed RNA-seq from HCT116 cells transfected with siCTRL or sip53 (SMARTpool of 4 siRNAs). Interestingly, *HKDC1* mRNA was significantly up-regulated (~2.25-fold) upon p53 knockdown (Figure 4A, 4B and Table S4). As expected, upon p53 knockdown, the mRNAs encoding p53 and its target genes *p21* and *ZMAT3* were strongly down-regulated (Figure 4A and 4B). We validated the observed up-regulation of *HKDC1* upon p53 knockdown by RT-qPCR and immunoblotting (Figure 4C and 4D). In these experiments, mRNA and/or protein levels of p53, p21 and ZMAT3 were markedly decreased upon p53 knockdown (Figure 4C and 4D). At a transcriptome-wide level, ~2850 genes were differentially expressed (p<0.05) upon p53 knockdown (Table S4 and Figure S4A). Intersection of the list of differentially expressed genes upon p53 knockdown with the ZMAT3-regulated genes identified 351 genes that were commonly upregulated and 425 genes that were commonly downregulated upon knockdown of p53 and ZMAT3 (Figure S4B and C). GSEA showed enrichment of glycolysis and the p53 pathway, for the genes upregulated and downregulated upon knockdown of p53 and ZMAT3, respectively (Figure S4D and E).

Since p53 knockdown resulted in increased HKDC1 expression, we next asked if inducing p53 would result in down-regulation of HKDC1. Indeed, when we looked at RNA-seq data, we observed ~40% reduction in *HKDC1* mRNA levels upon Nutlin treatment of siCTRL transfected HCT116 cells (Figure 4E and Table S3). As expected for a direct p53 target gene, upon p53 activation by Nutlin, *ZMAT3* and *p21* mRNAs were upregulated ~2.5- and ~6-fold, respectively; p53 mRNA levels didn’t change because Nutlin is known to specifically induce p53 protein levels (Figure 4E). At the protein level, p53 activation by Nutlin resulted in decreased HKDC1 levels and increased ZMAT3 and p21 levels in *ZMAT3*-WT cells (Figure 4F). In *ZMAT3*-KO cells, there was a modest increase in basal HKDC1 levels and a modest decrease upon Nutlin treatment (Figure 4F). Moreover, we generated doxycycline-inducible ZMAT3-FLAG-HA expressing HCT116 cells to overexpress ZMAT3. After 48 hours of doxycycline treatment ZMAT3-HA induction was confirmed by RT-qPCR and immunoblotting (Figure 4G and 4H). Furthermore, knockdown of p53 in these cells resulted in significant increase in both mRNA and protein levels of HKDC1 in untreated cells. Importantly, upon inducing ZMAT3 levels using doxycycline, the upregulation of HKDC1 mRNA and protein associated with p53 knockdown was not observed (Figure 4I and 4J). Collectively these data suggest that the p53/ZMAT3 signaling axis plays a critical role in the regulation of HKDC1 expression, where ZMAT3 operates downstream of p53 to inhibit HKDC1 expression.

### ZMAT3 inhibits HKDC1 transcription by interacting with the transcription activator JUN

ZMAT3 is an RNA-binding protein that regulates alternative splicing. We therefore hypothesized that ZMAT3 directly binds to the *HKDC1* pre-mRNA and regulates its splicing. However, when we looked at our ZMAT3 PAR-CLIP data^20^ we did not identify binding for ZMAT3 to *HKDC1* pre-mRNA (data not shown). Since the ZMAT3 protein has three C2H2-zinc-finger motifs it has the potential to bind to DNA (Supplementary Figure S5A)^32–34^. A recent report suggested ZMAT3 and other zinc finger proteins as DRBPs (DNA-RNA binding proteins)^33^. To examine if ZMAT3 binds to DNA and regulates HKDC1 expression directly at the transcriptional level we conducted ZMAT3-HA Cut&Run-seq and ChIP-seq in three biological replicates using anti-HA or anti-FLAG or anti-ZMAT3 antibody. However, we did not observe reproducible binding of ZMAT3 to the *HKDC1* locus or other regions of the genome in HCT116 cells (data not shown).

**Figure 5.**
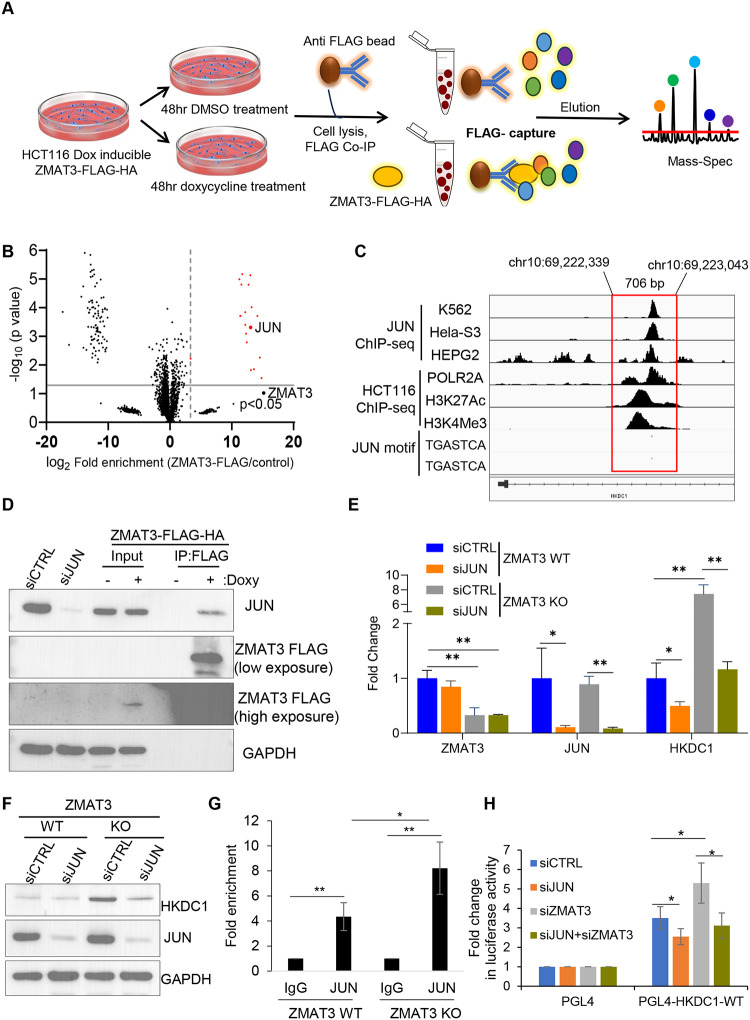
ZMAT3 inhibits HKDC1 transcription by interacting with the transcription factor JUN. **(A**) Schematic for identification of ZMAT3-FLAG interacting proteins by IP mass spectrometry from HCT116 cells expressing doxycycline-induced ZMAT3-FLAG-HA. **(B)** Volcano plot showing significantly enriched proteins (shown in red) identified by ZMAT3-FLAG IP followed by mass spectrometry, in presence or absence of doxycycline from ZMAT3-FLAG-HA HCT116 cells. JUN was strongly enriched in the ZMAT3-FLAG IPs. (**C**) IGV snapshot showing JUN, POLR2A, H3K27Ac and H3K3Me3 peaks at the *HKDC1* locus from the ENCODE project (accession from top to bottom: ENCSR000FAH, ENCSR000EDG, ENCSR000EEK, ENCSR000EUU, ENCSR661KMA and ENCSR333OPW). JUN binding motif (TGASTCA) is shown in blue (positive strand) and in red (negative strand). (**D**) Immunoblotting was performed using anti-FLAG beads and whole cell lysates from no doxy or doxycycline treated ZMAT3-FLAG-HA HCT116 cells. 10% of total cell lysate was used for input. GAPDH used as loading control. **(E, F)** *ZMAT3*- WT and KO HCT116 cells were transfected with CTRL siRNA or JUN siRNAs for 48 hr. The levels of *ZMAT3*, *JUN*, and *HKDC1* mRNAs or the corresponding proteins were measured by RT-qPCR (E) or immunoblotting from whole cell lysates (F). GAPDH was used as housekeeping gene control. **(G**) JUN ChIP-qPCR was performed in biological triplicates from HCT116 *ZMAT3*-WT and KO cells to determine enrichment of JUN at the *HKDC1* promoter. (H) Luciferase assays were performed in biological triplicates upon JUN or ZMAT3 knockdown alone or in combination using the *HKDC1* promoter reporter constructs. *p < 0.05, **p < 0.01

We therefore hypothesized that ZMAT3 inhibits HKDC1 expression by interacting with a specific transcription factor. To identify the proteins that interact with ZMAT3 we performed IPs using an anti-FLAG antibody followed by mass spectrometry from whole cell lysates of untreated or doxycycline-treated ZMAT3-FLAG-HA HCT116 cells (Figure 5A and S5B. After removing common contaminants (<10% in Crapome), this unbiased approach identified 21 ZMAT3-interacting proteins highlighted in red (Figure 5B and Supplemental Table S5). As expected, ZMAT3 was the most strongly enriched protein (~36,000-fold). Of note, due to low ZMAT3 levels in one of the controls, the p-value for ZMAT3 enrichment was not very significant (p=0.09) (Table S5).

Interestingly, the transcription factor JUN (c-Jun) was strongly enriched in the ZMAT3-FLAG pulldowns (~8,500-fold). JUN is a proto-oncogene and has previously been implicated in glucose metabolism^35,36^ and p53 function^37,38^. We next utilized publicly available JUN ChIP-seq data from various cell lines from ENCODE, with a focus on JUN binding at the HKDC1 promoter. Notably, in 3 cell lines, we found a JUN ChIP-seq peak having consensus JUN binding motif in the *HKDC1* intron 1. This peak coincided with ChIP-seq peaks for POLR2A, H3K27Ac and H3K4Me3 in HCT116 cells (Figure 5C). We validated the interaction between ZMAT3 and JUN proteins by ZMAT3-FLAG IP and immunoblotting (Figure 5D). Importantly, knockdown of JUN resulted in decreased *HKDC1* mRNA levels in ZMAT3- WT cells and rescued the elevated *HKDC1* mRNA and protein levels in ZMAT3-KO cells (Figure 5E and F). To determine if ZMAT3 inhibits JUN binding to the *HKDC1* locus we conducted ChIP-qPCR for JUN from *ZMAT3*-WT and KO cells. JUN exhibited significantly enhanced binding at the *HKDC1* intron 1 in *ZMAT3*-WT cells compared to the IgG control, and the enrichment of JUN to the *HKDC1* promoter was significantly increased in *ZMAT3*-KO cells, indicating that ZMAT3 inhibits the ability of JUN to bind to the *HKDC1* promoter. We next cloned a ~700 bp DNA fragment that encompassed the JUN and POLR2A binding peaks within the *HKDC1* promoter into the pGL4 basic luciferase reporter vector, designated as pGL4 HKDC1-promoter. Remarkably, knockdown of JUN in HCT116 cells resulted in a significant decrease in luciferase activity of the pGL4 HKDC1-promoter (Figure 5H). Conversely, ZMAT3 knockdown resulted in a marked increase in luciferase activity relative to control and simultaneous knockdown of ZMAT3 and JUN rescued the increase in reporter activity (Figure 5H).

**Figure 6:**
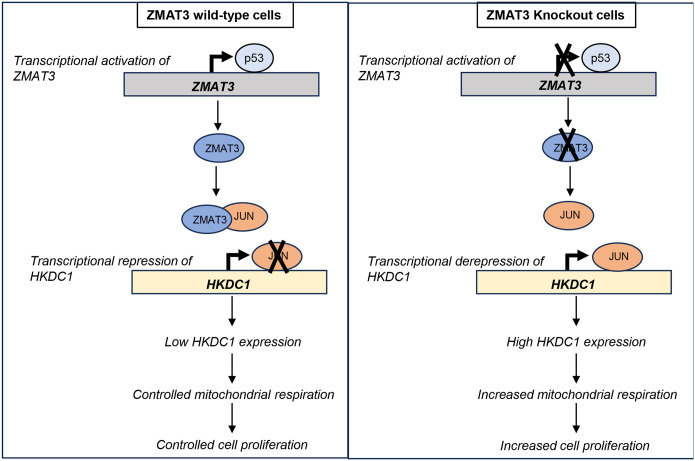
Schematic for ZMAT3-mediated regulation of HKDC1 expression and inhibition of mitochondrial respiration. In *ZMAT3*-WT cells p53 activates *ZMAT3* transcription, resulting in ZMAT3 protein binding to the transcription factor JUN. This inhibits the ability of JUN to bind to the *HKDC1* promoter, low *HKDC1* expression leading to controlled mitochondrial respiration and controlled cell proliferation. In *ZMAT3*-knockout cells, JUN actively binds to the *HKDC1* promoter and upregulates its expression resulting in increased mitochondrial respiration and increased cell proliferation.

Collectively, these data suggest that ZMAT3 plays a crucial role in controlling mitochondrial respiration and cell proliferation by suppressing *HKDC1* transcription. We propose a model according to which in *ZMAT3*-WT cells p53 drives ZMAT3 transcription, ZMAT3 protein binds to the HKDC1 transcription activator JUN thereby inhibiting its binding with *HKDC1* promoter which leads to transcriptional repression of HKDC1 thereby controlled mitochondrial respiration and controlled cell proliferation. In the absence of ZMAT3, JUN binds to the HKDC1 promoter and upregulates its expression, resulting in increased mitochondrial respiration and increased cell proliferation (Figure 6).

## Discussion

Recent studies suggest that ZMAT3 significantly contributes to the tumor suppressive effects of p53^17,18^. At the molecular level, ZMAT3 functions as an RNA-binding protein that acts as a key splicing factor and regulates mRNA stability^17,20^. Here, we unexpectedly found that the transcription of *HKDC1*, the gene that is most strongly upregulated at the protein level in ZMAT3-deficient cells, is indirectly repressed by ZMAT3 via interaction with the key transcription factor, JUN, thereby inhibiting JUN’s binding to the *HKDC1* promoter. Consistent with the well-established function of HKDC1 in glucose metabolism and mitochondrial respiration, we observed increased mitochondrial respiration upon upregulation of HKDC1 in ZMAT3-depleted cells and this phenotype was rescued upon concurrent knockdown of ZMAT3 and HKDC1, suggesting that HKDC1 is a key downstream effector of ZMAT3.

ZMAT3 has been known as a p53 target gene for more than two decades^39,40^, but its physiological function and role in tumor suppression are only beginning to be understood. ZMAT3 belongs to zinc finger family of proteins that play crucial role in regulating gene expression through specific recognition of DNA sequences^41^. Although they are primarily known for their involvement in transcription regulation, these proteins have also been found to interact with RNA and proteins^42,43^. Among them, ZMAT3 is a member of the ZMAT domain-containing family that has three zinc fingers of C2H2-type zinc fingers motif^44,45^. These domains play crucial roles transcriptional regulation by specifically binding to target molecules such as DNA and RNA. A recent study^33^ revealed ZMAT3 as a DRBP (DNA- and RNA-binding protein), but in our hands using Cut&Run-seq and ChIP-seq, we did not observe specific binding of ZMAT3-FLAG or endogenous ZMAT3 to DNA (data not shown). In these experiments Cut&Run-seq and ChIP-seq for p53 worked exceedingly well (data not shown), demonstrating that there was no technical flaw. It may be that ZMAT3 binding to DNA is cell-type specific, or upon DNA damage but this needs further investigation.

Our findings that ZMAT3 interacts with and inhibits binding of JUN to the HKDC1 locus provides mechanistic insights on how ZMAT3, without directly interacting with DNA regulates HKDC1 transcription. JUN is a protooncogene that plays a crucial role in both normal physiological processes and tumorigenesis by regulating cell proliferation, differentiation, senescence and metastasis^46–49^. It is a transcription factor function as a key component of AP-1 complex and promote RNA polymerase II mediated transcription of target genes^50^. It would be interesting to determine which ZMAT3 regulated genes are regulated via JUN. Future investigations are also needed to determine the domains of JUN that interact with ZMAT3 and what ZMAT3 does to JUN.

Because ZMAT3 regulates alternative splicing which can lead to changes in protein levels without altering mRNA levels, in this study we integrated quantitative proteomics data with RNA-seq data from ZMAT3-WT and isogenic ZMAT3-KO CRC cells. Furthermore, we integrated these data with RNA-seq from HCT116 cells upon ZMAT3 or p53 knockdown using siRNAs, to make sure that the findings from the isogenic cell lines were not restricted to a single KO clone and to determine the role of the p53/ZMAT3 axis in regulating these genes. This approach identified HKDC1 as the most strongly protein upon ZMAT3 depletion. HKDC1 is emerging as an important regulator of tumor progression and is frequently upregulated in several cancers including CRC^51–53^. Besides its hexokinase activity, HKDC1 interacts with the mitochondrial membrane and plays an essential role in mitochondrial function^26,27^. We further demonstrated that ZMAT3 suppresses glucose uptake and basal mitochondrial respiration by inhibiting HKDC1 expression leading to suppression of cell proliferation in CRC cells.

Our data also demonstrates that p53 negatively regulates *HKDC1* expression and this effect is ZMAT3-dependent, suggesting that the p53/ZMAT3/HKDC1 axis may be an important component of the p53 network, in the context of mitochondrial respiration and proliferation. The ability of p53 to induce cell cycle arrest and programmed cell death is important for tumor suppressor^54^. However, p53 has several other functions that recent data strongly implicate in tumor suppression, particularly regarding the control of metabolism such as glycolysis, mitochondrial respiration, and ferroptosis^55,56^. Metabolic reprogramming is a hallmark of cancer cells, which plays a pivotal role in cancer progression by providing energy and a wide variety of substrates for biosynthesis to support the rapid proliferation and survival of cancer cells^57–59^. p53 has been reported to play an important role in suppressing tumor development by regulating the expression and function of metabolic genes, directly *(GLUT1*^60^, *GLUT4*^60^, *PFKFB3*^61^ and *PFKFB4*^62^) or indirectly (*HK2*^63^*, HIF1α*^64^ and *G6PD*^65^). Our data uncovers HKDC1 as an indirect p53 target gene that is negatively regulated via ZMAT3. Collectively, our findings provide key insights into the diverse functions of ZMAT3 and their involvement in gene regulation in the p53 pathway.

## Materials and Methods

### Cell lines

The cell lines HCEC-1CT, HepG2, HCT116 and SW1222 were purchased from the American Type Culture Collection (ATCC). Cells were grown in DMEM medium (ThermoFisher scientific, Catalog no. 11995065), containing 10% Fetal Bovine Serum (FBS) (ThermoFisher scientific, Catalog no. 10082147) and 100 U/ml of penicillin and 0.1 mg/ml of streptomycin (ThermoFisher scientific, Catalog no. 15070063), at 37°C and 5% CO2. All cell lines were regularly screened for mycoplasma using the Venor^™^ GeM Mycoplasma Detection Kit (Millipore Sigma, Catalog no. MP0025–1KT).

### Targeted deletion of ZMAT3 using CRISPR/Cas9

To generate *ZMAT3*-KO clones, we used the PiggyBac CRISPR/Cas9 system adopted from the Zhang lab (Shalem et al., Science, 2014). Two sgRNAs flanking the p53RE in the *ZMAT3* intron 2 were designed and separately cloned in pENTR221 vector. These constructs were electroporated into 1×10^6^ parental HCT116 cells using Amaxa Cell Line Nucleofector Kit (Lonza, Catalog no. VCA-1005) along with pT3.5-Flag-Cas9, pCDNA-pB7, and pBSB-CG-LUC-GFP-(puro)(cre+) vectors. After two days, the cells were treated with 2 μg/ml puromycin (ThermoFisher scientific, Catalog no. A1113803) for three days. Single cells were then seeded in 96-well plates after puromycin selection to select *ZMAT3*-WT and *ZMAT3*-KO clones. The clones were harvested three weeks later and split into 24-well plates. Total RNA was isolated from each well of the 24-well plate, and ZMAT3 expression was measured by RT-qPCR normalized to *GAPDH*. Genomic DNA was extracted from individual clones in which ZMAT3 expression was profoundly decreased, and the DNA flanking the p53RE of ZMAT3 was PCR amplified and subjected to Sanger sequencing.

### Plasmids construction and lentivirus production

The pLVX-Puro-Tet-One vector from TaKaRa (631849) was used as a backbone to construct an expression vector containing ZMAT3–3xFLAG-2xHA. The vectors were transformed into DH5α cells (ThermoFisher scientific, Catalog no. 18265017), and the plasmids were purified using the Monarch plasmid miniprep kit (NEB T1010L). Lentiviruses were produced in 3×10^5^ 293T cells after co-transfection of 1 μg DNA with a third-generation lentivirus packaging system using Lipofectamine 2000 (Thermo Fisher Scientific Catalog no. 11668027). HCT116 cells were transduced at an MOI~1, and after 2 days, the cells were treated with 2 μg/mL puromycin (ThermoFisher scientific, Catalog no. A1113803) for 1 week.

Wild-type (pGL4-Basic-HKDC1-WT-promoter) POLR2A binding site present at the HKDC1 promoter construct were cloned into the pGL4-Basic vector (Promega, Catalog no. E6651) for luciferase assays. We used gene fragments (Twist Bioscience) at region chr10:69,222,339–69,223,043 (hg38) as HKDC1 JUN binding site, containing restriction sites from KpnI (NEB, Catalog no. R3142) and XhoI (NEB, Catalog no. R0146S) at 5′ and 3′, respectively. We digested the pGL4-Basic vector and DNA fragments with these enzymes, purified the products using the Monarch DNA gel extraction kit (NEB, Catalog no. T1020S) or QIAquick PCR purification kit (Qiagen, Catalog no. 28106), and ligated the inserts with the vector using T4 DNA ligase (NEB, Catalog no. M0202S)

### siRNA transfections

We used reverse transfection to deliver siRNAs with the use of Lipofectamine RNAiMAX Transfection Reagent (ThermoFisher Scientific Catalog no.13778075) and optiMEM (ThermoFisher Scientific Catalog no. 31985062) in HCT116, HCEC-1CT, HepG2 and SW1222 cells according to the manufacturer’s protocol. The final concentration of siRNA was 20 nM. For RT-qPCR and immunoblotting, we conducted two rounds of transfection. The second round was conducted 48 hours after the first transfection conducted for 72 hours. The HKDC1 transfection was conducted for only one round after the first transfection with siCTRL or siZMAT3. We used Negative Control siRNA (Qiagen, Catalog no. 1027281) as control. We used SMARTPool siRNAs against ZMAT3 (Horizon Discovery, Catalog no. L-017382-00-0005), siJUN (Horizon Discovery, Catalog no. L-003268-00-0005) and p53 (Horizon Discovery, Catalog no. L-003329-00-0005). For metabolic assays, we transfected cells using siRNAs against more than one target (e.g. siHKDC1 and siZMAT3) at 20nM each. HCT116 expressing the ZMAT3-FLAG-HA were transfected for 48 h and reseeded, the cells were treated with 2μg/ml doxycycline to induce the ZMAT3 expression.

### Luciferase assays

Cells were transfected with siRNAs for 48 hours, and then 1×10^5^ cells were reseeded for luciferase assay in 24-well plates. The next day, the cells were co-transfected with 250 ng of pGL4-ZMAT3-WT-promoter and 25 ng of pRL-TK (Promega, Catalog no. E2231) vectors, along with 20 nM of either Allstars negative control siRNA, siJUN or siZMAT3. Lipofectamine 2000 transfection reagent (ThermoFisher Scientific, Catalog no. 11668027) was used to carry out co-transfection, as per manufacturer’s protocol. After 2 days, firefly and Renilla luminescence from pGL4 and pRL-TK vectors, respectively, were measured using the dual-luciferase reporter assay system (Promega, Catalog no. E1910) according to the manufacturer’s protocol on EnSight Multimode plate reader (PerkinElmer). Firefly luminescence was normalized with Renilla luminescence for transfection efficiency.

### RNA extraction and RT-qPCR

Cells that were used to analyze the expression via RT-qPCR were washed with DPBS 1X (ThermoFisher Scientific, Catalog no. 14190250) after 48 hours of transfection. Then, they were lysed using 500 μl of TRIzol Reagent (ThermoFisher Scientific, Catalog no. 15596018) 48 hours after transfection, and RNA was extracted based on the manufacturer’s protocol. To prepare cDNA, 500 ng of RNA was reverse transcribed using iScript^™^ Reverse Transcription Supermix (Biorad, Catalog no. 1708841). In the qPCR reaction, 2.5 μl of diluted cDNA was combined with 5 μl of 2x FastStart Universal SYBR Green Master (Rox) (Millipore Sigma, Catlog no. 4913914001), 0.5 μM (final concentration) of each primer in a reaction with a final volume of 10 μl. The reactions were executed on StepOnePlus Real-Time PCR machine (Applied Biosystems), and *GAPDH* was used as a loading control. Finally, the relative expression was calculated using the 2-ΔΔCt method.

### Immunoblotting

For immunoblotting, cells were lysed using RIPA buffer (ThermoFisher Scientific, Catlog no. 89901). The lysates were sonicated three times for five seconds each at a power set of 50% using a VirTis VIRSONIC 100. Then, the lysates were centrifuged at 13000g for 10 minutes at 4°C, and the supernatant was collected. Pierce BCA Protein Assay Kit (ThermoFisher Scientific, Catalog no. 23225) was used for protein quantitation. For SDS-PAGE the gel was loaded with 20 to 50 μg of protein, transferred to a PVDF membrane using a BioRad semi-dry transfer apparatus and the membrane was blocked for 1 hour with TBST (Tris-Buffered Saline - 19.98 mM Tris, 136 mM NaCl and Tween 0.05%, pH 7.4) containing 5% milk. Anti-GAPDH antibody (1:6000 dilution; Cell Signaling, 5174S) was used for loading control. The following primary antibodies were used: anti-FLAG (1:1000 dilution; Sigma F1804), anti-p53 (DO-I) (1:1000 dilution; Santa Cruz Biotechnology sc-126), anti-ZMAT3 (1:500 dilution; Santa Cruz Biotechnology sc-398712), anti JUN (1:1000 dilution; Cell signalling, 9165S) and anti-HKDC1 (Proteintech, Catlog no: 25874–1-AP). After 1 hour of secondary antibody incubation at 1:5000 dilution, the membranes were developed using ECL^™^ Prime Western Blotting Detection Reagent (Fisher Scientific, Catalog no. RPN2232).

### Colony formation assays

One thousand ZMAT3-WT and isogenic ZMAT3-KO HCT116 cells were seeded in 6-well plates. After 12 days, the cells were fixed with ice-cold methanol for 15 minutes and stained with 0.5% crystal violet (prepared in 10% methanol) for 15 minutes. We used the ImageJ software (version 2.0.0-rc-43/1.52n) to analyze images of the colony coverage area.

### Incucyte Proliferation assays

To conduct the proliferation assays, 1000 cells were seeded per well in 96-well plates. The cells were then incubated on an Incucyte^®^ S3 Live-Cell Analysis Instrument and photographed every 6 hours for 4 days. The images were analyzed using the manufacturer’s software to measure % confluence over time.

### Cell viability assays

To determine cell viability, cells were incubated with cell counting kit-8 (Dojindo, Kumamoto, Japan) for 4 hour and absorbance at 450 nm was measured using a microplate reader Envision (PerkinElmer).

### Glucose uptake assays

siCTRL, siZMAT3 and siHKDC1 were transfected into HCT116, SW1222, and HepG2 cells in poly-l-lysine–coated white 96-well plates with opaque bottoms (Costar) and incubated at 37°C for 24 hours. After the incubation period, the growth medium was removed, and cells were washed twice with PBS to eliminate residual glucose. The Glucose Uptake-Glo kit (Promega, Catalog no. J1341) was used to measure cellular 2-deoxyglucose uptake, according to the manufacturer’s instructions. Glucose uptake was initiated by the addition of 1 mM 2-deoxyglucose for 10 minutes at 37°C following luminescence measurement in Envision Instrument.

### Metabolic flux assays

First, HCT116 cells were reverse transfected with siCTRL or siZMAT3 for 48 hours. After 48 hours, a second round of transfection was conducted in 24well plates Seahorse (Agilent Technologies Inc, Catalog no. 100777–004,) at a concentration of 3×10^4^ per 200μL per well. After 48 hours cells were washed twice with 500mL of XF DMEM Medium, pH 7.4 (Agilent Technologies Inc, Catlog no. 103575–10) containing 1mM pyruvate, 2mM of glutamine and 10 mM of glucose (Agilent Technologies Inc, Catalog no. 103578–100, 103579–100, 103577–100). Eventually cells were incubated 45 minutes in a non-CO2 incubator prior to the assays. Meanwhile drugs from Mito Stress Test Kit (Agilent Technologies Inc, Catalog no. 103015–10) and Glycolytic Rate Assay Kit (Agilent Technologies Inc, Catalog no. 103344–100) were prepared in XF DMEM Medium, pH 7.4 containing 1mM pyruvate, 2mM of glutamine and 10mM of glucose. For the Mito Stress Test Kit the working drug solutions concentration were Oligomycin 1.5μM, FCCP 1.0μM and Rot/AA 0.5μM. For the glycolytic Rate Assay Kit the working drug solutions concentration were Rot/AA 0.5μM and 2-DG 50mM. After the 45min of incubation, the plates were loaded into the Seahorse Analyzer and the commercial protocols for the drugs distribution were used. Immediately after the assays, media was removed from wells and cells frozen for later protein concentration measurement. Protein concentrations were measured with PierceTM BCA Protein Assay kit (ThermoFisher Scientific, Catalog no. 23225) and used to normalize Seahorse results.

### Gene set enrichment analysis

GSEA using MSigDB Hallmark gene sets was performed on the website of the Broad Institute (http://www.gsea-msigdb.org/gsea/msigdb/annotate.jsp). The proteins that showed significant (p<0.05) upregulation upon ZMAT3-KO were used as input.

### Co-immunoprecipitation

ZMAT3-FLAG coimmunoprecipitations were performed using FLAG M2 antibody-coated magnetic beads (Sigma-Aldrich Catalog no. M8823). 5 × 107 doxycycline treated/untreated HCT116 ZMAT3-FLAG-HA cells lysed in IP lysis buffer (10 mM Tris/Cl pH 7.5, 150 mM NaCl, 0.5 mM EDTA, 0.5 % NP40) supplemented with 1 mM PMSF, complete protease inhibitor cocktail (Roche). Lysates were incubated for 30 min at 4°C with periodic mixing and clarified by centrifugation at maximum speed for 10 min at 4°C. Equal amounts of protein lysates were incubated with washed M2 beads overnight at 4°C with constant rotating. Beads were magnetically separated from supernatant and washed four times with IP Wash buffer (10 mM Tris/Cl pH 7.5, 150 mM NaCl, 0.05 % NP40, 0.5 mM EDTA). Bound proteins were eluted with FLAG elution buffer supplemented with 125 μg/ml 3xFLAG peptide [F4799; Sigma-Aldrich]). Equal volumes of eluates were boiled at 100°C for 5 min in Laemmli sample buffer and then centrifuged at maximum speed for 5 min at RT. Five percent of the total cell lysate was used as input, and proteins were detected by immunoprecipitation. For IP mass spectrometry, samples were processed after washing without elution step.

### ChIP-qPCR

ChIP-qPCR was carried out using an Active Motif ChIP-IT express kit (catalog no. 53008) following the manufacturer’s instructions. In brief, 5 × 10^7^ ZMAT3-WT and ZMAT3 KO HCT116 cells grown in 15-cm plates were cross-linked with 1% formaldehyde, scraped, lysed, and then sheared. The size of the chromatin was verified on a 1% agarose gel. Chromatin was immunoprecipitated at 4°C overnight with 1μg of anti-JUN and IgG antibody. The IP material was washed, eluted, and reverse cross-linked overnight at 65°C. ChIP DNA was column purified (Qiagen PCR purification, catalog no. 28104) and analyzed by qPCR. ChIP-qPCR primers were designed based on the genomic sequence of the JUN peaks found in the HKDC1 intron-1.

### RNA-seq and analysis

RNA-seq was performed in biological triplicates from HCT116 ZMAT3-WT and ZMAT3-KO cells. Total RNA was isolated using the RNeasy Plus mini kit (Qiagen, Catalog no. 74134) following the manufacturer’s instructions. We used the Illumina Stranded mRNA Ligation library Kit. We used 450ng of total RNA as the input to an mRNA capture with oligo-dT coated magnetic beads. The mRNA is fragmented, and then a random-primed cDNA synthesis is performed. The resulting double-strand cDNA is used as the input to a standard Illumina library prep with end-repair, adapter ligation and PCR amplification being performed to give you a sequencing ready library. The final purified product is then quantitated by qPCR before cluster generation and paired end sequencing on NovaSeq 6000 SP 200 cycles run.

The Illumina bcl2fastq2.20 was used to demultiplex and convert binary base calls and quality scores to Fastq format. The sequencing reads were trimmed for adapters and low-quality bases using Cutadapt (v1.18). The trimmed reads were mapped to human reference genome (hg38) and Gencode annotation v30 using STAR aligner (v2.7.0f) with two-pass alignment option. RSEM (v1.3.1) was used for gene and transcript quantification based on GENCODE annotation.

### Mass Spectrometry Sample Preparation

For total protein identification, cell pellets were suspended in 8M urea buffer supplemented with protease and phosphatase inhibitors (Roche). All samples were transferred to 2 ml TissueLyser tubes containing 5 mm steel balls on ice. Samples were lysed in a TissueLyser (Qiagen) for 2 × 2 mins with chilling in-between at −20°C for 2–3 mins. Lysates were centrifuged in a microcentrifuge at 12,500 rpm for 15 min (4°C). Supernatants were transferred to new tubes and proteins concentrations were measured using BCA method (Thermo). For downstream processing, 200 ug of each sample were reduced with 10 mM DTT at 56°C for 1 hour and alkylated with 20 mM iodoacetamide at room temperature for 30 minutes in the dark. Following alkylation, samples were diluted 4-fold with 50 mM triethylammonium bicarbonate (TEAB) to reduce the urea concentration to 2 M and digested with trypsin (40:1) at 37°C overnight. Digested peptides were desalted using C18 columns and lyophilized. Peptide concentrations were measured using colorimetric BCA peptide assay (Thermo). For TMT labeling, 100 ug of digested peptides were labeled with the TMTpro 16-plex reagent at room temperature (dark) for 1 hour. Reactions were stopped by addition of 5% hydroxyl amine and incubation at room temperature (dark) for 15 mins. Following labeling, peptide samples were pooled and lyophilized. Lyophilized pooled samples were suspended in 0.1% TFA and fractionated using high pH Reverse-Phase Peptide Fractionation Kit (Thermo) with nine elution buffers containing 0.1% Triethylamine and 10%, 12.5%, 15%, 17.5%, 20%, 22.5%, 25%, 50%, and 75% acetonitrile, respectively. Fractions were lyophilized separately.

For ZMAT3-FLAG interacting protein identification, IP samples were solution digested with trypsin using S traps (Protifi), following the manufacturer’s instructions. Briefly, proteins were denatured in 5% SDS, 50 mM triethylammonium bicarbonate (TEAB) pH 8.5. They were next reduced with 5 mM Tris(2-carboxyethyl)phosphine (TCEP) and alkylated with 20 mM iodoacetamide. The proteins were acidified to a final concentration of 2.5% phosphoric acid and diluted into 100 mM TEAB pH 7.55 in 90% methanol. They were loaded onto the S-traps, washed four times with 100 mM TEAB pH 7.55 in 90% methanol, and digested with trypsin overnight at 37 °C. Peptides were eluted from the S-trap using 50 mM TEAB pH 8.5; 0.2% formic acid in water; and 50% acetonitrile in water. These elutions were pooled and dried by lyophilization.

### Mass Spectrometry Analysis

Dried peptides were resuspended in 5% acetonitrile, 0.05% TFA in water for mass spectrometry analysis on an Orbitrap Exploris 480 (Thermo) mass spectrometer. The peptides were separated on a 75 μm × 15 cm, 3 μm Acclaim PepMap reverse phase column (Thermo) at 300 nL/min using an UltiMate 3000 RSLCnano HPLC (Thermo) and eluted directly into the mass spectrometer. For analysis, parent full-scan mass spectra acquired at 120,000 FWHM resolution and product ion spectra at 45,000 resolutin with a 0.7 m/z isolation window. Proteome Discoverer 3.0 (Thermo) was used to search the data against the human database from Uniprot using SequestHT and with INFERYS rescoring. The search was limited to tryptic peptides, with maximally two missed cleavages allowed. Cysteine carbamidomethylation and TMT pro modification of lysine and peptide N-termini were set as a fixed modification, with methionine oxidation as a variable modification. The precursor mass tolerance was 10 ppm, and the fragment mass tolerance was 0.02 Da. The Percolator node was used to score and rank peptide matches using a 1% false discovery rate. TMT quantitation was performed using the Reporter Ions Quantifier nodes with correction of the values for lot-specific TMT reagent isotopic impurities.

### TCGA COAD gene expression

Gene expression and clinical data of COAD were obtained using the TCGA biolinks (v2.16.0) package in R (v4.0.0). The GDC query function was used to query samples by setting the data category to “gene expression,” data type to “gene expression quantification,” platform to “Illumina HiSeq,” file type to “normalized results,” and experimental strategy to “RNA-seq.” Data were then downloaded using GDC download followed by GDC prepare to obtain normalized gene expression values.

### Statistical analysis

The statistical analysis for all data was performed using at least three replicates. The significance of statistical analyses was tested using two-tailed Student’s t-test when comparing two groups or two-way ANOVA. Pearson coefficient correlation was used for correlation analysis with the TCGA COAD study.

## Supplementary Material

1

## Data Availability

The RNA-seq data from ZMAT3-WT vs ZMAT3-KO HCT116 cells and from siCTRL vs sip53 from HCT116 have been deposited to the GEO (https://www.ncbi.nlm.nih.gov/geo/query/acc.cgi?acc=GSE280756).
