# Supplementary material for: p53-induced RNA-binding protein ZMAT3 inhibits transcription of a hexokinase to suppress mitochondrial respiration
Source: bioRxiv. 2025 May 13:2025.05.12.653341. Preprint. [Version 1] doi: 10.1101/2025.05.12.653341 (PMC12087986; doi:10.1101/2025.05.12.653341)

bioRxiv preprint doi: https://doi.org/10.1101/2025.05.12.653341; this version posted May 13, 2025. The copyright holder for this preprint (which was not certified by peer review) is the author/funder. This article is a US Government work. It is not subject to copyright under 17 USC 105 and is also made available for use under a CC0 license.

Figure S1. Extended results for Figure 1.

(A) Immunoblotting for ZMAT3, p53, p21 and GAPDH from *ZMAT3*-WT and *ZMAT3*-KO HCT116 cells with or without Nutlin treatment (24 hr). GAPDH served as the loading control. **(B)** Incucyte live cell proliferation assays were performed from *ZMAT3*-WT and *ZMAT3*-KO HCT116 cells. **(C)** Volcano plot for the differentially expressed genes identified by RNA-Seq from *ZMAT3*-WT and *ZMAT3*-KO HCT116 cells. Significantly expressed genes are indicated in red ($p<0.05$). (**D**) Most significantly enriched pathways identified by GSEA from the top 500 significantly upregulated genes ($p<0.05$) from *ZMAT3*-KO vs *ZMAT3*-WT RNA-seq.

bioRxiv preprint doi: https://doi.org/10.1101/2025.05.12.653341; this version posted May 13, 2025. The copyright holder for this preprint (which was not certified by peer review) is the author/funder. This article is a US Government work. It is not subject to copyright under 17 USC 105 and is also made available for use under a CC0 license.

Figure S2. Extended results for Figure 2.

(A) Volcano plot for the differentially expressed genes identified by RNA-Seq performed after transfection of HCT116 cells with siCTRL or siZMAT3 for 72 hr. Significantly expressed genes are indicated in red (p<0.05). **(B)** GSEA analysis was performed for the top 500 significantly upregulated genes (p<0.05) in ZMAT3 knockdown HCT116 cells identified by RNA-seq. (**C, D**) Venn diagram of showing comparisons of the indicated RNA-Seq data sets. 1,023 significant upregulated (C) and 1,042 downregulated (D) differentially expressed genes were shared between the *ZMAT3*-KO/*ZMAT3*-WT and siZMAT3/siCTRL comparisons. **(E)** Most significantly enriched pathways in the GSEA for the top 500 genes commonly upregulated genes (p<0.05) in ZMAT3-KO vs *ZMAT3*-WT and siZMAT3 vs siCtrl comparisons from the RNA-seq data. **(F, G)** ZMAT3 and HKDC1 mRNA levels were determined in CRC patient samples in the TCGA COAD cohort from p53-WT (wild-type) and p53-Mutant CRC patient samples.

Figure S3. Extended results for Figure 3.

Non-mitochondrial oxygen consumption in ZMAT3 and/or HKDC1 knockdown in HCT116 cells. Values are the average of four independents experiments.

Figure S4. Extended results for Figure 4.

(A) Volcano plot shows the differentially expressed genes from the RNA-Seq from sip53 and siCTRL transfected groups in HCT116 cells. Significantly expressed genes are shown in red (p<0.05). (**B, C**) Venn diagram and GSEA analysis for the genes commonly upregulated in RNA-Seq upon p53 knockdown and ZMAT-KO HCT116. (**D, E**) Venn diagram and GSEA analysis for the genes commonly downregulated in RNA-Seq upon p53 knockdown and *ZMAT*-KO HCT116.

Figure S5. Extended results for Figure 5.

(A) Schematic of full-length ZMAT3 protein showing three zinc finger motifs. **(B)** Immunoblot from 10% input and FLAG immunoprecipitation from doxycycline inducible ZMAT3-FLAG-HA HCT116 cell lysates treated with/without doxycycline for 48 hr.

was not certified by peer review) is the author/funder. This article is a US Government work. It is not subject to copyright under 17 USC 1
and is also made available for use under a CC0 license.

Figure S1

A

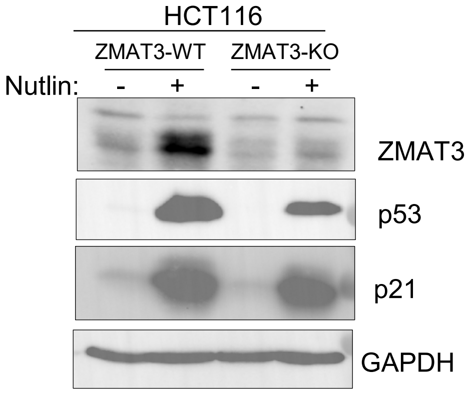

B

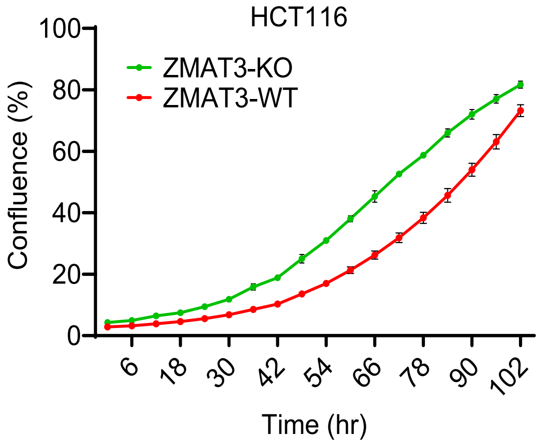

C

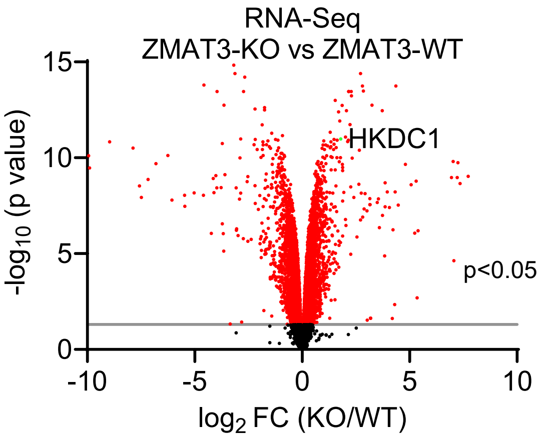

D

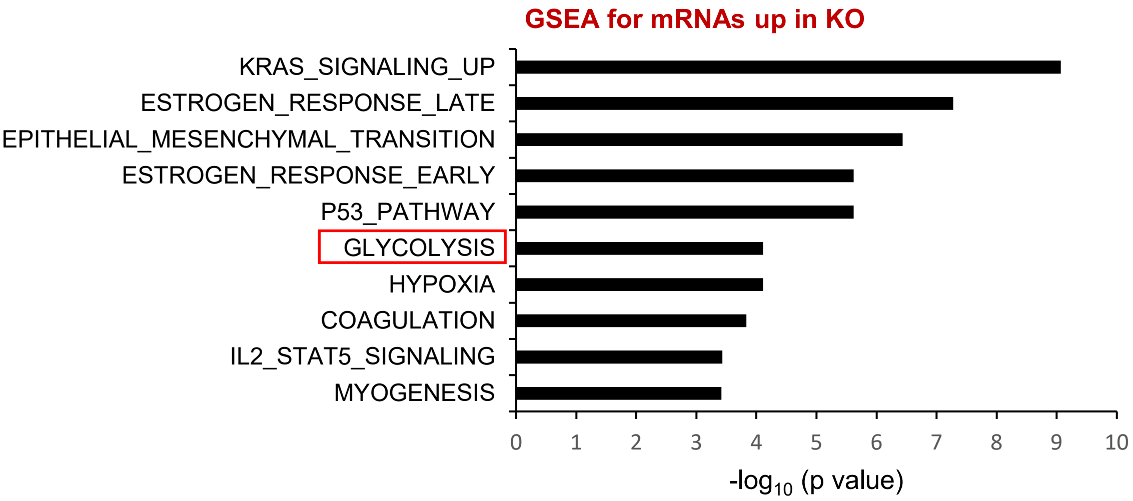

was not certified by peer review) is the author/funder. This article is a US Government work. It is not subject to copyright under 17 USC 1
and is also made available for use under a CC0 license.

Figure S2

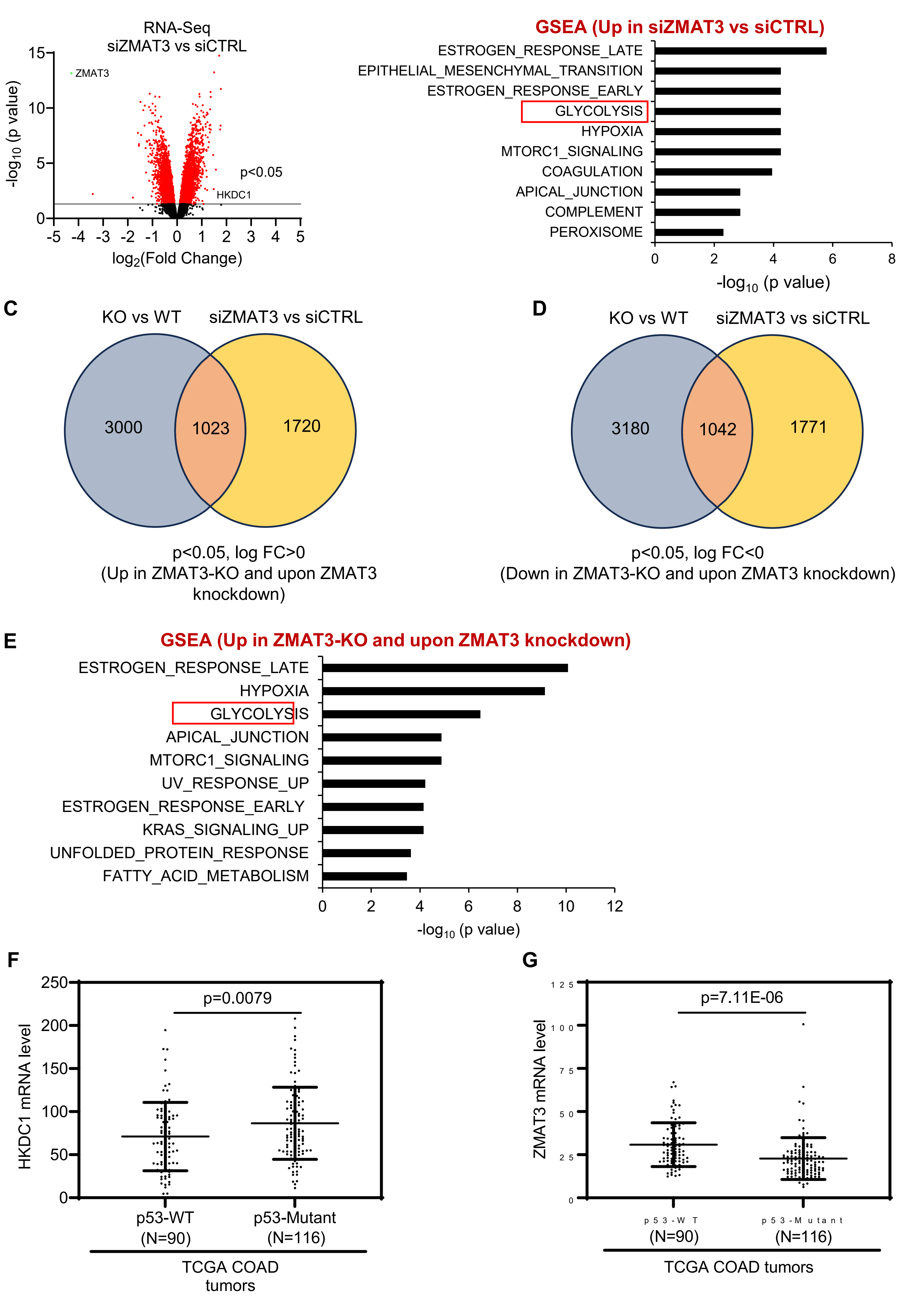

was not certified by peer review) is the author/funder. This article is a US Government work. It is not subject to copyright under 17 USC 1
and is also made available for use under a CC0 license.

Figure S3

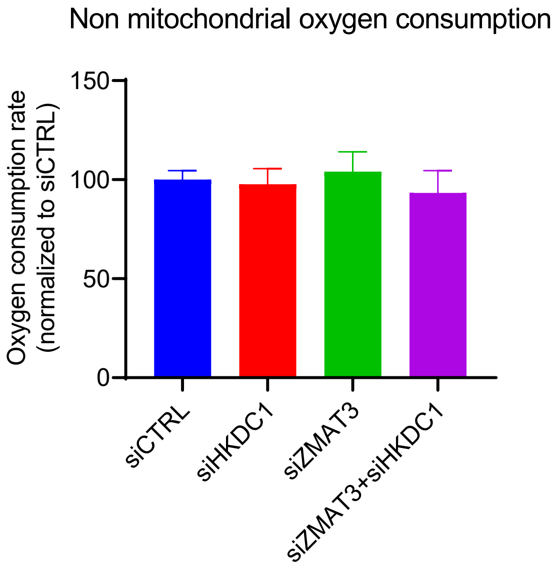

was not certified by peer review) is the author/funder. This article is a US Government work. It is not subject to copyright under 17 USC 1
and is also made available for use under a CC0 license.

Figure S4

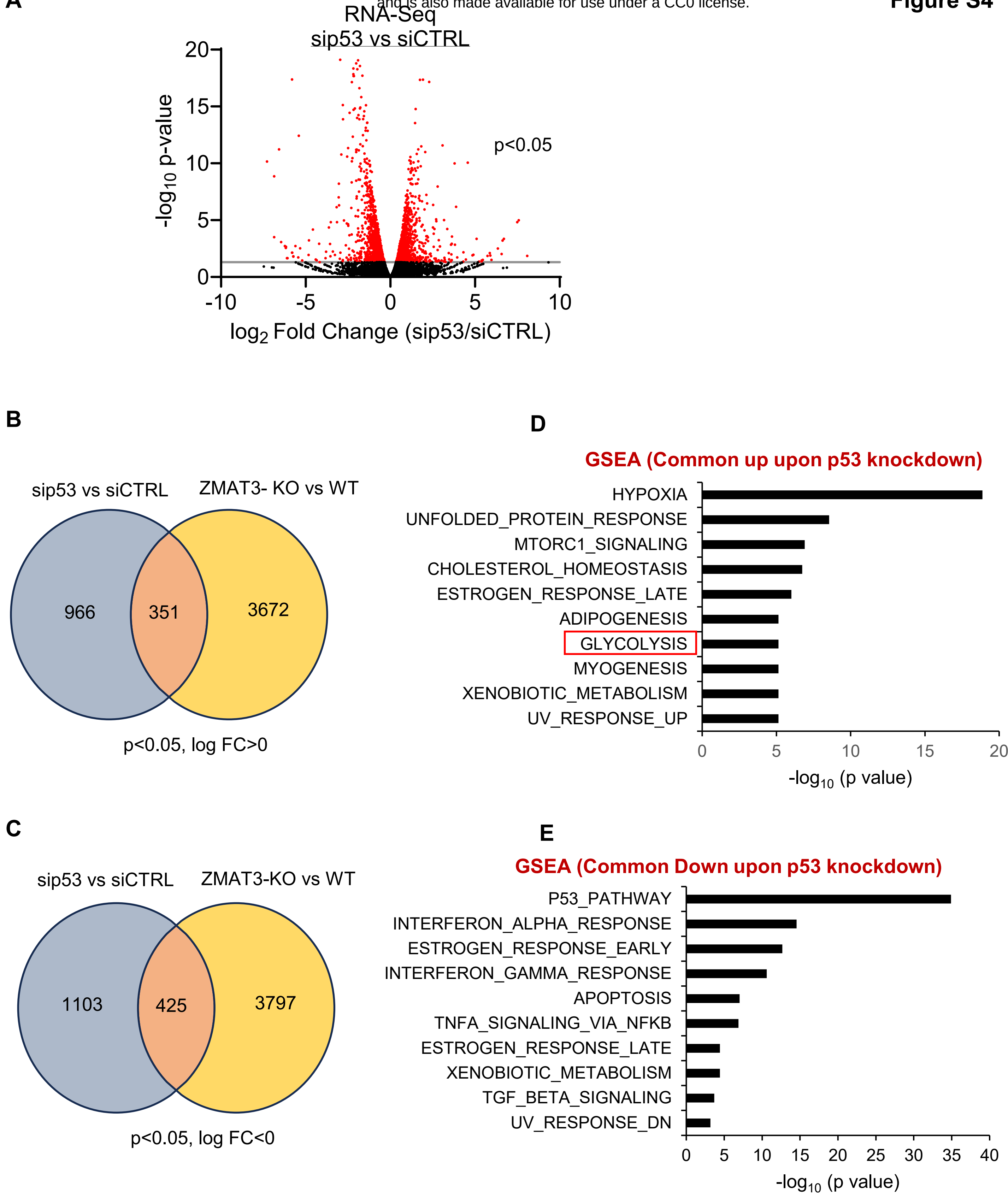

was not certified by peer review) is the author/funder. This article is a US Government work. It is not subject to copyright under 17 USC 1
and is also made available for use under a CC0 license.

Figure S5

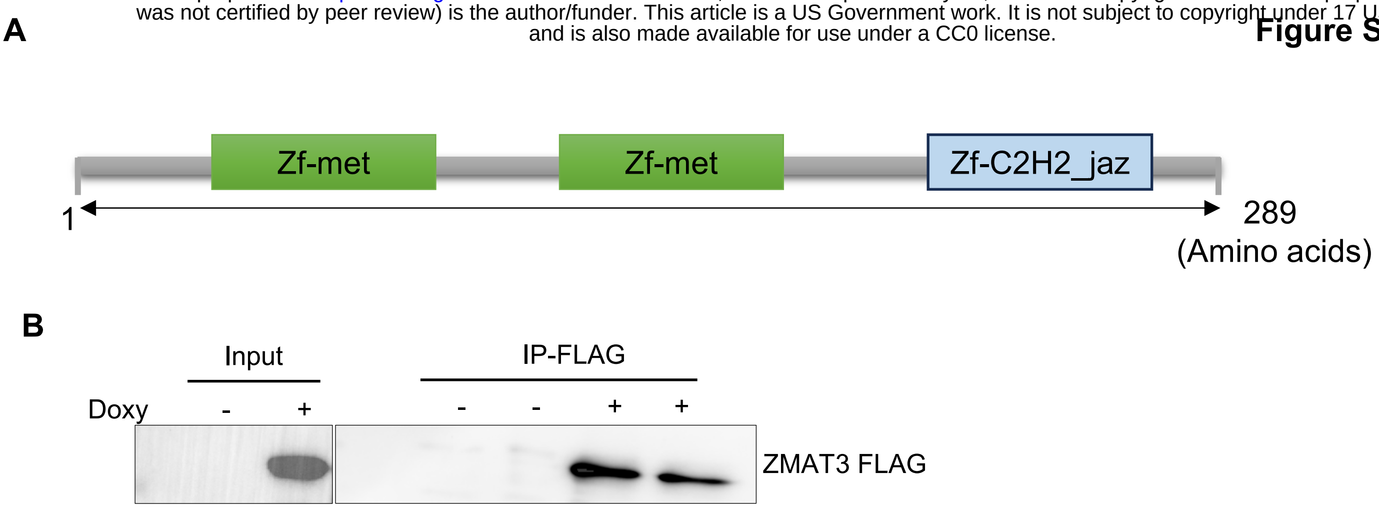

Supplement: 1 [file NIHPP2025.05.12.653341V1-supplement-1.pdf]
